# Estimation of moisture ratio for apple drying by convective and microwave methods using artificial neural network modeling

**DOI:** 10.1038/s41598-021-88270-z

**Published:** 2021-04-28

**Authors:** Vali Rasooli Sharabiani, Mohammad Kaveh, Roozbeh Abdi, Mariusz Szymanek, Wojciech Tanaś

**Affiliations:** 1 Department of Biosystem Engineering, Faculty of Agriculture and Natural Resources, University of Mohaghegh Ardabili, Daneshgah Street, 56199-11367 Ardabil, Iran; 2 Department of Agricultural, Forest and Transport Machinery, University of Life Sciences in Lublin, Głęboka 28, 20-612 Lublin, Poland

**Keywords:** Statistics, Electronics, photonics and device physics

## Abstract

Two different drying methods were applied for dehydration of apple, i.e., convective drying (CD) and microwave drying (MD). The process of convective drying through divergent temperatures; 50, 60 and 70 °C at 1.0 m/s air velocity and three different levels of microwave power (90, 180, and 360 W) were studied. In the analysis of the performance of our approach on moisture ratio (MR) of apple slices, artificial neural networks (ANNs) was used to provide with a background for further discussion and evaluation. In order to evaluate the models mentioned in the literature, the Midilli et al. model was proper for dehydrating of apple slices in both MD and CD. The MD drying technology enhanced the drying rate when compared with CD drying significantly. Effective diffusivity (D_eff_) of moisture in CD drying (1.95 × 10^−7^–4.09 × 10^−7^ m^2^/s) was found to be lower than that observed in MD (2.94 × 10^−7^–8.21 × 10^−7^ m^2^/s). The activation energy (Ea) values of CD drying and MD drying were 122.28–125 kJ/mol and 14.01–15.03 W/g respectively. The MD had the lowest specific energy consumption (SEC) as compared to CD drying methods. According to ANN results, the best R^2^ values for prediction of MR in CD and MD were 0.9993 and 0.9991, respectively.

## Introduction

Apple (Malus domestica Borkh.) is one of the oldest fruits known to mankind and has grown to nourish it. It is one of the most important horticultural products in the world, and countries such as China, the United States, Turkey, Poland, India, the Russian Federation and Iran are considered as major apple producers. Apples, like many other fruits, have a high water content (80–85% on the wet basis (w.b.)). Apple is rich in vitamins, minerals and fiber and is usually consumed raw, but it is used in many foods (especially desserts) and beverages^1–3^. Drying, in addition to being a way to increase the shelf life of foods, is known as a way to increase the value added of food products. Removing water from a product under controlled conditions reduces the moisture content of the food to a certain extent, which lessens the activity of enzymes, the rate of undesirable chemical changes and microbial growth. Also, the decrease in moisture is accompanied by a reduction in volume and weight, which is one of the important factors for transportation and maintenance^4^. Throughout the decades, hot air drying method has been one of the most long-established technologies in the food industries. The process of hot air drying includes both the heat and mass transfer while the water is provided by the agricultural products through diffusion. However the total energy of this diffusion goes hand in hand with air temperature, time and air velocity^5^. One of the methods that has been given a lot of attention during the last decade is drying using microwave radiation. Microwave beams are electromagnetic beams with a long wavelength of 2450 MHz. During the passing of these waves from the tissue of matter, polar molecules, such as water and salts, vibrate, and this vibration causes the microwave energy to be converted into heat. Unlike other methods of drying, in which heat should penetrate from the surface to depth, in this method heat is produced in the tissue of the food itself and it is prevented from damaging the superficial parts of the food^6,7^. Different methods are used to reduce the moisture content of fruits and vegetables. Izli and Isik^8^ used microwave, convective, and microwave-convective dryers to dry tomatoes. They showed that microwave-convective dryers require less time to dry tomatoes. Seremet et al.^9^ investigated effect of different drying methods (Convective and Convective—microwave dryer) on weight loss and rehydration of sliced pumpkin. Drying of sorbus fruits by convective (50 °C and 70 °C at air velocity of 0.3 m/s) and microwave (90, 160 and 350 W) were studied in order to determine the drying behaviors. The results showed that the temperature of 50 °C and the microwave power of 90 W had the slightest variations in color. Also, the lowest specific energy consumption were 0.69 kWh/kg and 37.07 kWh/kg respectively at 70 °C and 350 W^10^. The correlation of the unpredictable input and output process parameters interconnection follows the stimulated computing approach named Artificial Neural Network (ANN) ^11^. ANNs are capable of modeling nonlinear and complex systems with a large number of input and output data. The ability to predict a neural network is completely dependent on its structure (type of activation function, number of layers and number of hidden layer neurons)^12,13^. In recent years, methods based on ANNs have been used to predict the moisture content of many food and agriculture products during the drying process, including green peas, tomatoes, corn and pomegranate seeds^14–17^. In this research, the neural network modeling method was used to estimate the moisture ratio of apple slices during drying in microwave and hot air dryer. The results of this model are compared with the results of mathematical modeling to determine its effectiveness. Also, moisture diffusion coefficient, activation energy, specific energy consumption and color changes were also determined for apple slices.

## Material and methods

### Sample preparation

Apple was supplied from one of apple orchards of Ardabil city, Iran, in September 2019, according to the two national standards (ISIRI 2011 and ISIRI 2007)^18,19^. Generally, apple samples of uniform sizes were selected. The apple fruit were cleaned and stored in a refrigerator at 4 ± 1 °C. The premature and spoiled apple was separated manually. The initial MC of apple slices was measured by oven drying method. Apple slices to the nearest 40 g (4 mm thickness and 36 mm diameter) in triplicate samples were dehydrated at 70 ± 1 °C for 24 h^20^. Apple fruit with average initial MC of 87 ± 3% (w. b) was selected for drying material.

### Experimental procedure

#### Convective dryer

Convective drying (CD) was conducted by using laboratory drying oven (BF55E; FG Co., Iran). The velocity of the air approaching to the apple samples was measured by an anemometer (Lutron AM-4202; Electronic Enterprise Co., Taipei, Taiwan) with ± 0.1 m/s accuracy and the average air velocity was 1.2 ± 0.02 m/s. Electrical heating unit of this dryer equipped with PT100 thermometer sensor and PID controller with ± 0.1 °C accuracy. Average humidity and air temperature of ambient air during CD dryer were 30% and 26 °C, respectively.

#### Microwave dryer

A fully programmable microwave oven (Panasonic NN-CD997S Microwave Oven) with maximum output of 1000 W was utilized for this study. The microwave oven has the capability of operating at different microwave output powers, 90, 180, 270, 360, 450, 540, 630, 720, 900 and 1000 W. The MD drying area is 462 mm, 242 mm and 412 mm inner size and includes a 380 mm diameter rotary glass plate at the oven base. The microwave output power and processing time was set fully by using digital control panel of microwave oven.

### Experimental setup

#### Determination of moisture ratio

Drying curves may be represented in different ways; MC (wet and dry base) versus time, drying rate versus time, or drying rate versus MC. The MC of apple was calculated by using Eq. (1)^21^; Eq. (2) was used for calculating the moisture ratio of apple slices^22^:

1 $$ MC = \frac{{\left( {\left( {W_{x} - W} \right) - W_{y} } \right)}}{{W_{y} }} $$

2 $$ MR = \frac{{\left( {M_{t} - M_{e} } \right)}}{{\left( {M_{0} - M_{e} } \right)}} $$

It should be noted that due to the insignificant value of $M_{e}$ in comparison with $M_{t}$ and $M_{0}$, it can be saved, Therefore Eq. (2) can be simplified to Eq. (3)^23^:

3 $$ MR = \frac{{M_{t} }}{{M_{0} }} $$

#### Mathematical modelling of drying curves

The models listed in Table 1 were used for mathematical modeling drying kinetics of apple slice in MD and CD. To compare the data to each model, curve expert was used for curve fitting. This software has linear and nonlinear regression models and various interpolation methods. In order to select the suitable drying kinetics descriptor, the statistical parameters of $R^{2}$, $RMSE$ and $\chi^{2}$ were used. Finally, the drying model with maximum $R^{2}$ and minimum $RMSE$ and $\chi^{2}$ was selected as the appropriate model for describing drying kinetics. The mentioned statistical parameters are defined by the following equations^24,25^:

4 $$ R^{2} = 1 - \frac{{\sum\limits_{i = 1}^{N} {[MR_{\exp ,i} - MR_{pre,i} ]^{2} } }}{{\sum\limits_{k = 1}^{N} {\left[ {\frac{{\sum\limits_{k = 1}^{n} {MR_{pre,i} } }}{N} - MR_{pre,i} } \right]^{2} } }} $$

5 $$ \chi^{2} = \frac{{\sum\limits_{i = 1}^{N} {\left( {MR_{\exp ,i} - MR_{pre,i} } \right)^{2} } }}{N - z} $$

6 $$ RMSE = \left[ {\frac{1}{N}\sum\limits_{i = 1}^{N} {(MR_{pre,i} - MR_{\exp ,i} )^{2} } } \right]^{\frac{1}{2}} $$

**Table 1:** Mathematical empirical drying models given by various authors for the drying curves.

Models	Equation	References
Midilli et al.	$MR = a\exp ( - kt^{n} ) + bt$	^27^
Page	$MR = \exp ( - kt^{n} )$	^28^
Logistic	$MR = a/(1 + b\exp (kt))$	^29^
Two-term	$MR = a\exp ( - k_{0} t) + b\exp ( - k_{1} t)$	^30^
Logarithmic	$MR = a\exp ( - kt) + c$	^31^

Exponential equation form of Eq. (2) can be used as follows^26^:

7 $$ MR = \frac{{M_{t} - M_{e} }}{{M_{0} - M_{e} }} = \exp ( - kt^{n} ) $$

#### Effective moisture diffusivity

Mass transfer during food drying is a complex process involving various mechanisms such as molecular penetration, movement in capillary tubes, and liquid penetration in the porous materials, penetration of vapor in air pores and hydrodynamic flow, or surface propagation. Moisture penetration is one of the most important factors controlling the drying process. When different mechanisms are effective in transmitting, it is difficult to examine each mechanism and measure the mass transfer rate in each one. Hence, in such processes, the description of D_eff_ is used and its concept is described by the Fick’s second law as follows^32^:

8 $$ \frac{\partial M}{{\partial t}} = D_{eff} \nabla^{2} M $$

Calculation of D_eff_ using the Fick’s second law is a tool for describing the drying process and possible mechanisms for the transfer of moisture within food products. The analytical solution of Fick’s law is as follows^33^:

9 $$ MR = \frac{{M_{t} - M_{e} }}{{M_{0} - M_{e} }} = \frac{8}{{\pi^{2} }}\sum\limits_{n = 0}^{\infty } {\frac{1}{{(2i^{{}} + 1)^{2} }}\exp \left( { - \frac{{(2i + 1)^{2} \pi^{2} }}{{4L^{2} }}D_{eff} t} \right)} $$

Therefore, Eq. (9) can be written in simpler form as Eq. (10):

10 $$ MR = \frac{8}{{\pi^{2} }}\exp \left( { - \frac{{\pi^{2} D_{eff} t}}{{4L^{2} }}} \right) $$

The coefficient $K_{1}$ is calculated by plotting the curve ln (*MR*) versus time, in accordance with Eq. (11) as follows^34^:

11 $$ K_{1} = \left( {\frac{{D_{eff} \pi^{2} }}{{4L^{2} }}} \right) $$

#### Activation energy

Dependence of the D_eff_ with temperature is shown using the Arrhenius equation (Eq. 12). Activation energy of the CD dryer ($E_{a(c)}$) was determined by plotting the D_eff_ curve versus absolute air temperature reversal^35^.

12 $$ D_{eff} = D_{0} \exp \left( {\frac{{E_{a(c)} }}{{R_{g} T_{a} }}} \right) $$

The linear form of Eq. (12) can be obtained by applying the logarithms as:

13 $$ \ln (D_{eff} ) = \ln (D_{0} ) - \left( {\frac{{E_{a(c)} }}{{R_{g} }}.\frac{1}{{T_{a} }}} \right) $$

$K_{2}$ can be obtained by plotting $\ln (D_{eff} )$ versus $\frac{1}{{T_{a} }}$:

14 $$ K_{2} = \frac{{E_{a(c)} }}{{R_{g} }} $$

Linear regression analyses were used to fit the equation to the experimental data to obtain correlation coefficient (R^2^).

The activation energy for MD dryer ($E_{a(m)}$ (W/g)) was calculated by using a correlation between D_eff_ and ($\frac{m}{P}$) is taken into account^36^:

15 $$ D_{eff} = D_{0} \exp \left( { - \frac{{E_{a(m)} m}}{P}} \right) $$

$E_{a(m)}$ may be accomplished using one of several methods as follows:

16 $$ \ln (D_{eff} ) = \ln (D_{0} ) - \left( {\frac{{E_{a(m)} }}{P}.m} \right) $$

Following plotting of $\ln (D_{eff} )$ versus (1/P), $K_{3}$ is calculated for the microwave as follows:

17 $$ K_{3} = \frac{{E_{a(m)} }}{P} $$

#### Specific energy consumption

The specific energy consumed during the drying process, which is the amount of energy used to evaporate one kilogram of water from the product, was obtained using Eq. (18) under MD drying method^37^:

18 $$ SEC_{mic} = \frac{{P_{mic} t_{1} }}{{m_{w} }} $$

Specific energy consumption ($SEC_{con}$) of apple slice in CD approach was measured through the Eq. (19) as follows^38,39^:

19 $$ SEC_{con} = (C_{pa} + C_{pv} h_{a} )Qt_{2} \frac{{(T_{in} - T_{am} )}}{{m_{v} V_{h} }} $$

#### Color

Three color schemes, including RGB, CMYK and Lab, are used to determine the color of food. The Lab model is often used for food color research studies. L demonstrates brightness in the range 0–100, and two colored components ( − 120 to + 120) including a (greenness to redness) and b (blueness to yellowness). The color parameters of apple slice were measured using digital portable colorimeter (CR-10-PLUS, Konica Minolta Co, Japan), appropriate test method based on CIELAB. Total color changes ($\Delta E$) was calculated using Eq. (20). All color changes were obtained with averaging in six replicates samples^40,41^:

20 $$ \Delta E = [(\Delta L^{*} )^{2} + (\Delta b^{*} )^{2} + (\Delta a^{*} )^{2} ]^{0.5} $$

#### ANN

**Figure 1:**
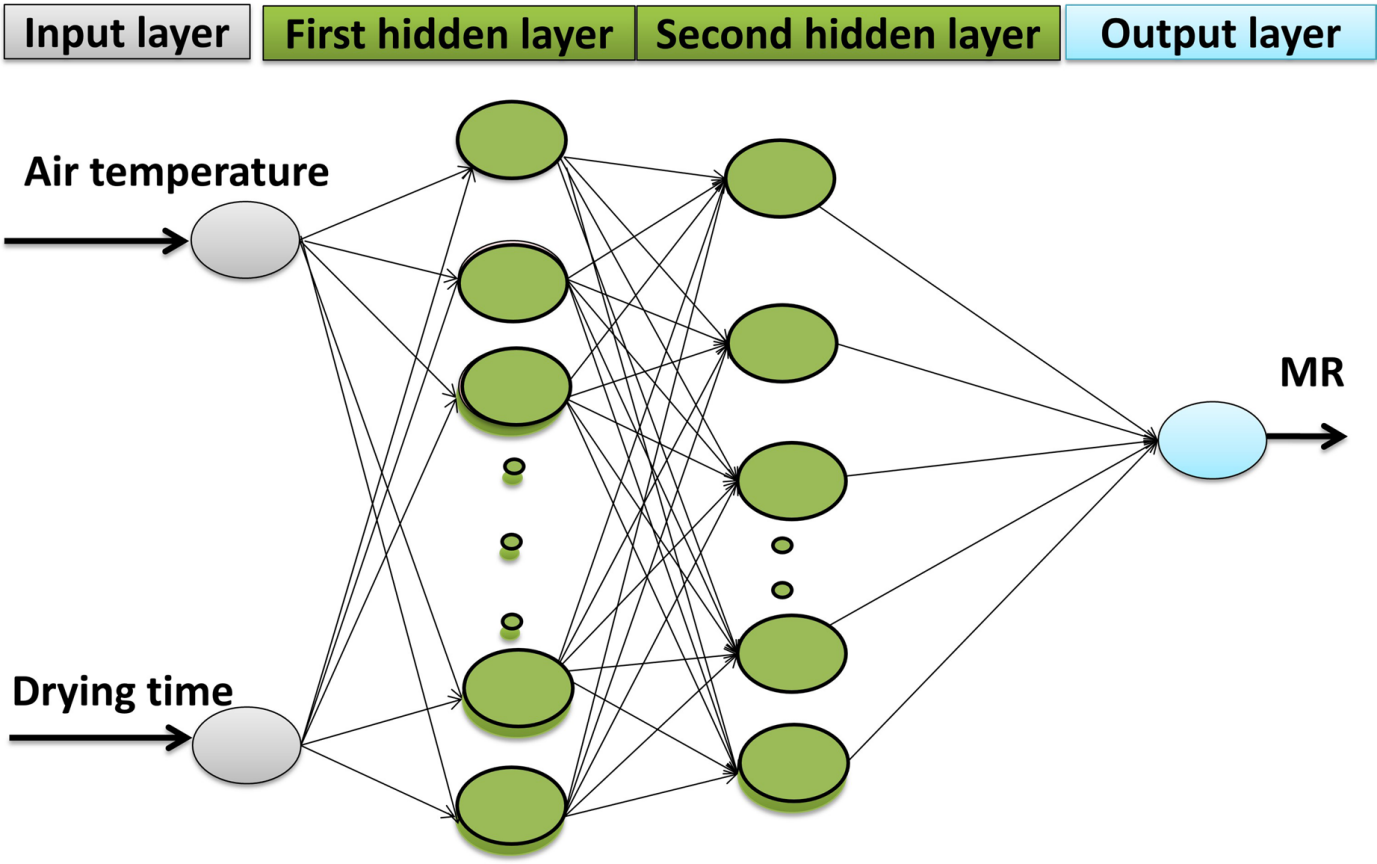
Four-layer ANN inputs and output structure used in this study.

ANN was used for modeling the drying process of apple slice in microwave and hot air dryer to predict MR by using Matlab software. In this research, the Levenberg–Marquard optimization method was used to teach the network. The inputs for ANN model are drying time, and drying chamber inlet air temperature, and the output is MC variations of apple slice. Figure 1 shows ANN inputs and output structure with two hidden layers.

##### Convective dryer

Apple slice drying experiments were done at 50, 60 and 70 °C set temperature. The two input parameters had applied in the experiments with CD dryer. The MR values were derived. Networks with two neurons in the input layer (air temperature and drying time) and one neuron in the output layer (MR) were designed. In this part, the total data of, moisture ratio (163 data) for artificial neural networks were used. In the first group, 70% (115 data) were taken for training phase and in the second group 30% (48 data) for testing, chosen randomly from the set of 163 data.

##### Microwave dryer

Applying the two inputs in all experiments, the MR values obtained for different conditions. Networks with two neurons in input layer (microwave power and drying time) and one neuron in output layer (MR) were designed. About 70% (49 data) of the all experimental data (70 data) were separated for network training to find suitable structure. Prior to training the neural network, input data normalized to it. The purpose of normalizing is to convert data between zero and one. Therefore, the following equation was used for normalization^42^:

21 $$ U_{n} = \frac{{U_{R} - U_{\min } }}{{U_{\max } - U_{\min } }} $$

In order to evaluate the accuracy and performance of the developed models of artificial neural networks, the statistical criteria of the coefficient of determination (R^2^), root mean square error (RMSE) and mean absolute error (MAE) were used. The mentioned statistical parameters calculated using the following equations^43^:

22 $$ MSE = \frac{1}{mq}\sum\limits_{p = 1}^{m} {\sum\limits_{i = 1}^{q} {(S_{ip} - T_{ip} )^{2} } } $$

23 $$ R^{2} = 1 - \frac{{\sum\limits_{k = 1}^{m} {\left[ {S_{k} - T_{k} } \right]} }}{{\sum\limits_{k = 1}^{m} {\left[ {S_{k} - \frac{{\sum\limits_{k = 1}^{n} {S_{k} } }}{n}} \right]} }} $$

24 $$ MAE = \frac{100}{n}\sum\limits_{k = 1}^{m} {\left| {\frac{{S_{k} - T_{k} }}{{T_{k} }}} \right|} $$

## Results and discussion

### Drying characteristics (convective and microwave drying kinetics)

**Figure 2:**
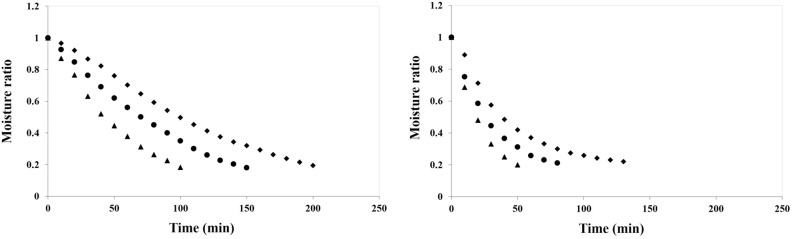
Moisture ratio versus drying time for apple slice under different drying: (**a**) air temperature ((♦) 50 °C, (●) 60 °C, (▲) 70 °C); (**b**) microwave powers ((♦) 90 W, (●) 180 W, (▲) 360 W).

Changes in MR of apple slice with drying time at different air temperatures 50, 60, and 70 °C and air velocity 1 m/s were presented in Fig. 2a. The drying experiments of apple slices continued until the MC of the samples reached about 0.20 (w.b.) in both drying methods. As can be seen in Fig. 2a, increase of air temperature from 50 to 70 °C causes a decrease on final product drying time, which is consistent with the results of Beigi^44^ and Kaleta et al.^45^. In the process of CD drying, increasing air temperature from 50 to 70 °C resulted to increase in mass transfer, reduce process time and energy consumption^46^. Drying time for apple slice in air velocity 1 m/s, were 200, 150 and 100 min at 50, 60 and 70 °C, respectively. Kara and Doymaz^46^ reported that the drying times of apple pomace at air velocity 1 m/s for four air temperatures 50, 60, 70 and 80 °C. An increment in air temperature caused also a decrease in drying time at apple pomace. Beigi^44^ reported that air temperature had a shorter effect on drying time in Hot air drying of apple slices at 1.5 m/s air velocity 50, 60 and 70 °C. Effect of air temperature (50, 65, 80 and 90 °C), three levels of drying product thickness (3,5,and 7 mm), engine load levels (25, 50, 75, and 100%), and air velocity (1 m/s) on moisture ratio of apple slices in combined heat and power (CHP) dryer have been investigated by Samadi et al.^47^. With increasing of air temperature in the tested range, the amount of moisture removed from apple slices increased.

Changes in MR of apple slice with drying time in MD dryer at different microwave power (90, 180 and 360 W) were shown in Fig. 2b. As shown in Fig. 2b, it can be seen that the rate of water loss in MD method was higher than CD, due to the electromagnetic heating effect of MD in drying food products^48^. Also, with higher microwave power, more heat generated within the sample created a larger vapor pressure difference between the center and the product surface. Thus accelerated the interior moisture migration and increased surface water evaporation^49^.

The times of the drying process in MD were 50, 80 and 130 min at 360, 180 and 90 W, respectively. The results showed that with increasing microwave power, the drying time had a downward trend. Similar results were obtained for drying crops in a microwave dryer such as pomegranate arils^50^, mushroom, tomatoes^8^ and broccoli stalk slice^51^.

In order to mathematical modeling of apple slice drying kinetics in the CD dryer, five commonly mathematical models for thin layer products were used (Table 1). For all CD drying experiments (50, 60 and 70 °C air temperature and 1 m/s air velocity), determination coefficient ($R^{2}$), root mean square error (RMSE), and reduced Chi square ($\chi^{2}$) values ranged between 0.9932–0.9999, 0.0172–0.0845 and 0.0003–0.0468, respectively. From Table 2, Midilli et al. model had the highest $R^{2}$ (0.9994–0.9999) and the lowest $RMSE$ (0.0194–0.0274) and $\chi^{2}$ (0.0004–0.0041) values. Therefore, the Midilli et al. drying model was achieved as appropriate one for describing drying behaviors of apple slices. Chayjan et al.^52^ tested five empirical models (Midilli et al., Demir et al., Logistics, Logarithmic and Wang and Singh) in their research for continuous band drying of eggplant slices and claimed that all models described well drying kinetics at studied air temperatures, air velocities and infrared power. Midilli et al. model was chosen as the best model for describing the moisture ratio of the sour cherry. Torki-Harchegani et al.^53^ examined the kinetics of drying lemon slices in CD dryer at 50, 60 and 75 °C air temperature. Their results showed that the drying temperature had a significant effect on the drying time and drying rate. Among the models used, the Midilli et al. model was proposed as the best model for prediction MR of lemon slices.

**Table 2:** The statistical comparison for prediction drying of apple slices in CD dryer.

Model	$R^{2}$			$\chi^{2}$			$RMSE$		
	50 °C	60 °C	70 °C	50 °C	60 °C	70 °C	50 °C	60 °C	70 °C
Midilli et al.	0.9999	0.9998	0.9998	0.0003	0.0009	0.0010	0.0172	0.0211	0.0221
Page	0.9998	0.9998	0.9996	0.0007	0.0011	0.0014	0.0282	0.0681	0.0741
Logistics	0.9993	0.9991	0.9989	0.0039	0.0059	0.0103	0.0268	0.0302	0.0398
Two-term	0.9966	0.9941	0.9932	0.0222	0.0401	0.0468	0.0629	0.0886	0.0845
Logarithmic	0.9998	0.9994	0.9990	0.0018	0.0028	0.0031	0.0224	0.0240	0.0251

The results of the fitting of apple slices drying data in MD method with different mathematical models were presented in Table 3. For all microwave drying experiments, $R^{2}$, $RMSE$ and $\chi^{2}$ values ranged between 0.9966–0.9999; 0.0188–0.0421 and 0.0005–0.0291. Midilli et al. model had the highest $R^{2}$ (0.9999) and the lowest $RMSE$ (0.0188) and $\chi^{2}$ (0.0005) values. Therefore, Midilli et al. model was proposed as the best model for drying apple slice in MD. Darvishi et al.^54^ dried white mulberry in microwave drying at 100, 200, 300, 400 and 500 W power levels and applied the experimental data to five thin layer models where Lewis, Henderson and Pabis, Page, Wang and Singh, and Midilli et al. models, the Page model gives the highest $R^{2}$ (0.999) and lower $RMSE$ (0.009) and $\chi^{2}$ (0.00009) values. Ganesapillai, Murugan & Singh^55^ used 100, 180, 300, 450, 600 and 900 W power levels for dehydration of Ginger rhizomes and to determine the drying characteristics of sample, Henderson, Page, Logarithmic, Wang and Singh, Diffusion, Verma, Two-term exponential, Midilli models tested. Diffusion model gave the best results (maximum values $R^{2}$ = 0.99958, minimum values $RMSE$ (0.00429) and $\chi^{2}$ (0.00019).

**Table 3:** The statistical comparison for prediction drying of apple slices in MD dryer.

Model	$R^{2}$			$\chi^{2}$			$RMSE$		
	90 W	180 W	360 W	90 W	180 W	360 W	90 W	180 W	360 W
Midilli et al.	0.9999	0.9996	0.9994	0.0005	0.0021	0.0027	0.0188	0.0249	0.0251
Page	0.9994	0.9991	0.9995	0.0057	0.0084	0.0148	0.0279	0.0313	0.0410
Logistics	0.9981	0.9967	0.9967	0.0077	0.0194	0.0272	0.0304	0.0501	0.0596
Two-term	0.9976	0.9974	0.9968	0.0116	0.0243	0.0324	0.0359	0.0564	0.0618
Logarithmic	0.9968	0.9979	0.9966	0.0018	0.0051	0.0291	0.0231	0.0268	0.0421

### Effective moisture diffusivity

**Figure 3:**
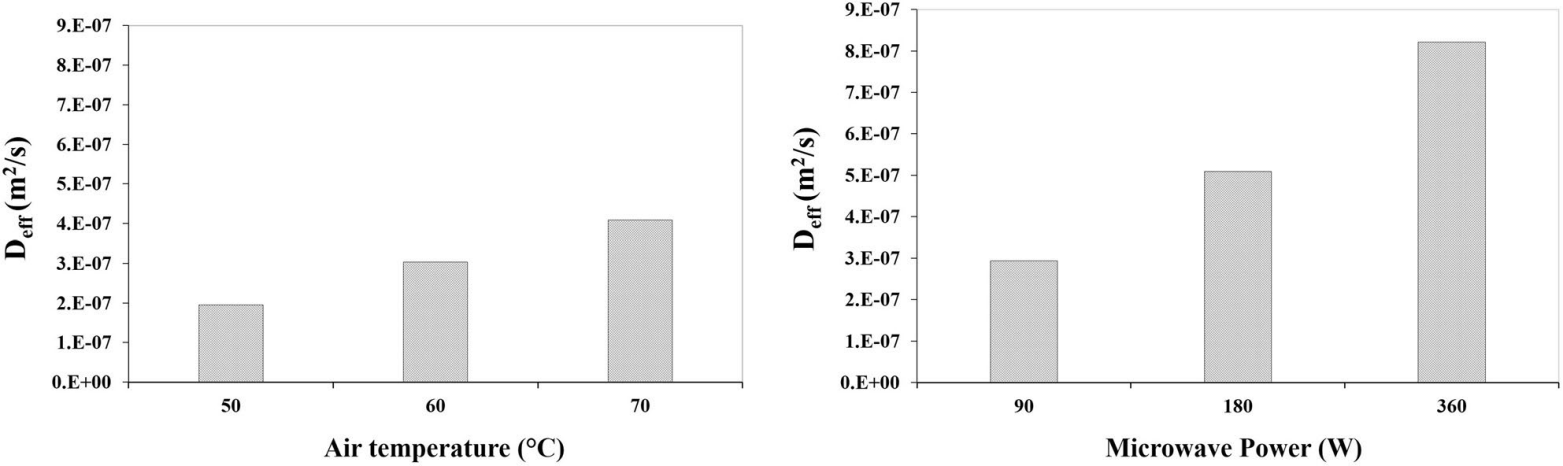
Effective moisture diffusivity ($D_{eff}$) for drying of apple slice in different: (**a**) air temperature, (**b**) microwave power.

Effective moisture diffusivity values (*D*_*eff*_) of apple slice at different dryer calculated by Eq. (11). The Reported *D*_*eff*_ values were within the general range of 10^–7^ to × 10^–12^ m^2^/s for agricultural product and food materials^29,56^. In CD drying, minimum *D*_*eff*_ value (1.95 × 10^–7^ m^2^/s) belonged to pretreated apple slice of at air temperature 40 °C, and maximum value (4.09 × 10^–7^ m^2^/s) belonged to apple slice at air temperature 70 °C. Obtained values were demonstrated in Fig. 3a. The results indicated direct correlation between *D*_*eff*_ and temperature. Increasing the air temperature was accompanied by an increase in *D*_*eff*_ and a reduction in the drying time. Beigi^44^ estimated as 7.03 × 10^–10^ to 1.08 × 10^–9^ m^2^/s for apple in CD dryer at 50 °C and 70 °C, 1.5 m/s air velocity. The values of *D*_*eff*_ are comparable with the reported values of 1.73 × 10^–10^ to 4.40 × 10^–10^ m^2^/s for apple pomace at 50 to 80 °C and air velocity of 2 ± 0.1 m/s in CD dryer^44^, 6.97 × 10^–11^ to 2.38 × 10^–10^ m^2^/s for quince slices drying in CD dryer^57^ and 3.27 × 10^–9^ to 1.23 × 10^–8^ m^2^/s consideration for broccoli slices with air temperatures of 45–70 °C and air velocities of 2 m/s in CD dryer^58^.

The values of *D*_*eff*_ for microwave dryer are presented in Fig. 3b. In microwave drying, minimum value (2.94 × 10^-7^ m^2^/s) belonged to apple slice which had a microwave power level of 90 W; maximum value (8.21 × 10^–7^ m^2^/s) belonged to apple slice that had a microwave power level of 360 W. According to the results the values of *D*_*eff*_ in MD were higher than CD. Also, the microwave power can accelerate the water molecules present in the apple slice to evaporate faster, thus providing a faster decrease of the apple slice MC and the corresponding higher value of D_eff_^59^.

Similar results for the amount of *D*_*eff*_ in MD dryers are provided by other authors for fruits and vegetables. For example: *D*_*eff*_ values for ginger rhizomes was obtained ranged from 20.24 × 10^–12^ to 9.8 × 10^–11^ m^2^/s at 100–900 W^53^, *D*_*eff*_ values for bamboo shoot slices increased from 4.15 × 10^–10^ to 22.83 × 10^–10^ m^2^/s at different power levels ranging from 140 to 350 W in microwave dryer^60^ and *D*_*eff*_ of mulberry increased with increasing microwave power. It varied from 1.06 × 10^–8^ to 3.45 × 10^–8^ m^2^/s at five microwave powers of 100, 200, 300, 400 and 500 W^54^.

### Activation energy

During the drying process, the highest values of activation energy for CD and MD methods were obtained 125 kJ/mol and 15.03 W/g, respectively (Table 4). The air temperature and microwave power were important factors influencing the *D*_*eff*_ and E_a_. By increasing the temperature and microwave power, the activation energy was reduced as the result of mass transfer and more moisture loss of apple slice. The obtained results are in line with the stated values for hot air drying of cherry tomato 31.99 and 36.21 kJ/mol for the raw and blanched cherry tomatoes^5^, and MD drying of kiwi slices (17.96–21.38 W/g)^61^.

**Table 4:** The estimated activation energy in convective and microwave dryer.

Air temperature (**°**C)	50 °C	60 °C	70 °C
Ea (kJ/mol)	125	124.44	122.28

Microwave power (W)	90 W	180 W	360 W
Ea (W/g)	15.03	14.50	14.01

### Specific energy consumption (convective and microwave)

**Figure 4:**
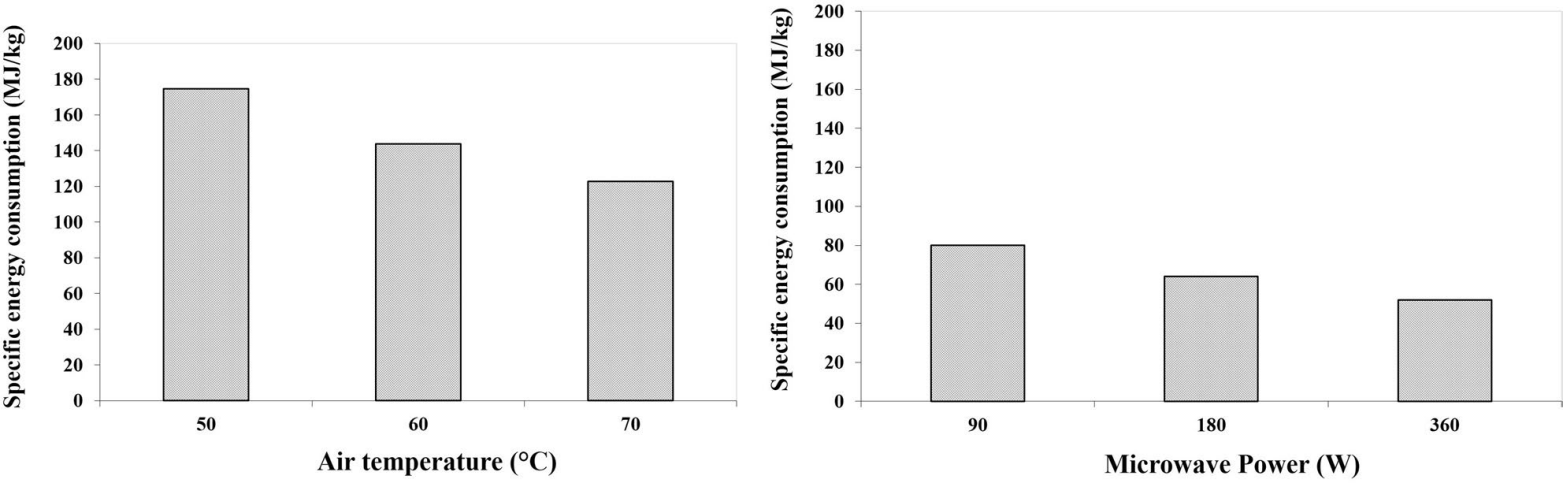
Variations of specific energy consumption for drying process of apple slice in different: (**a**) air temperature, (**b**) microwave power.

Figure 4a shows the SEC of drying process of apple slice in CD dryer. In this study, the SEC was obtained in the range of 122.77 to 174.67 MJ/kg. According to the results, the highest and lowest energy values were consumed in the process of drying apple slices at 50 and 70 °C, respectively. As shown in Fig. 4a, the increase in the air temperature of the dryer chamber from 50 to 70 °C continuously reduces SEC. In spite of lowering the specific heat of the air at higher temperatures, because of the significant reduction in the process time at these temperatures, the increase in the air temperature of the dryer chamber decreases the amount of energy consumed by the process. The values of SEC are comparable with the reported values of 74.73 MJ/kg mentioned for fluidized bed drying of rough rice^62^, average SEC for potato in CD dryer was obtained 3.491 MJ/kg^63^.

As shown in Fig. 4b, during the drying process of apple slices in MD dryer, the SEC at 90 and 360 W microwave power were obtained 80.05 and 52.03 (MJ/kg), respectively. In other words, the ratio of highest to lowest value of SEC was 1.53. According to the results, with the increase in microwave power, the SEC dropped significantly. The reduction in SEC at higher microwave power in MD method is due to the effect of its volumetric heating, which reduces the drying time^39,64^. Advantages such as shorter drying times and lower SEC were the key drivers for the further development of the microwave drying technique^65^. The minimum value of SEC for drying of green pepper were obtained to be 7.20 MJ/kg at microwave power 360 W^37^ and the maximum and minimum values of SEC in MD drying for tomato were obtained to be 350 and 8.4 Wh, respectively at range 90–600 W^66^.

### Color (convective and microwave)

**Figure 5:**
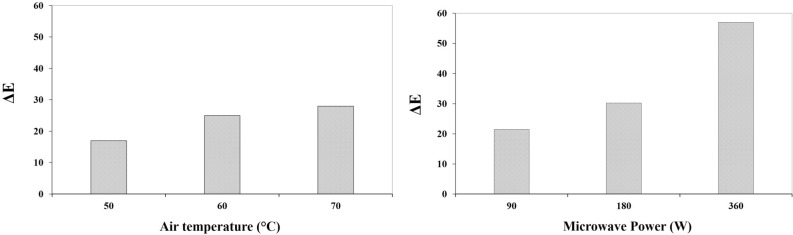
Color change values of apple slices in different (**a**) air temperature, (**b**) microwave power.

Color is one of the most important qualitative properties of fresh, processed food and its marketing. As shown in Fig. 5a, color variations ($\Delta E$) at different temperatures of 50, 60 and 70 °C are shown in CD dryer. The most and the least amount of color variations ($\Delta E$) occurred at temperatures of 70 and 50 °C, respectively. According to the results, with the increase in air temperature of the dryer, the amount of color changes ($\Delta E$) increased due to the decrease in MC in apple slice and the shrinkage process^67^. During the drying process, oxidation occurs and as a result of this oxidation, the intensity of the color decreases. The color change ($\Delta E$) of pomegranates arils (6.80–14.16) at hot air dryer were reported by Horuz and Maskan^50^. The minimum color change (9.316) was obtained at air velocity of 2 m/s, air temperature (60 °C) and belt linear speed of 2.5 mm/s, while the maximum color change was 18.24 observed at 1.5 m/s air velocity, air temperature 40 °C and belt linear speed (mm/s) of 10.5 mm/s, for terebinth^38^.

As shown in Fig. 5b, color changes in MD dryer increased by increasing microwave power from 90 to 180 W. The microwave power and process time are the effective factors influencing the color change in the MD dryer. Due to the heat, the chlorophyll green pigments may turn into pheophytin, which has a brownish color. The change in the color of the pigments can be due to the effect of heat on heat-sensitive compounds such as carbohydrates, proteins and vitamins, which also causes color change during the drying process^68,69^.

### ANN

#### Convective dryer

Table 5 presents the best results for combining CFBF and FFBF networks with different topologies and activation functions to predict the MR of apple slices in CD method. According to Table 5, we can get the best performance of the FFBF network, which with the topology 2-10-10-1, along with the TAN, TAN and PUR threshold function and LMA (Levenberg–Marquardt algorithm) for training neural network, has the best result through the three-layer and four-layer topologies. The selected topology created the highest level of correlation (0.9993 for train and 0.9994 for test) and the lowest values of MAE and MSE achieved were 0.0047 for train, 0.0041 for test and 0.00044 respectively, for output variables. Tavakolipour et al.^70^, the MR of zucchini were predicted by using ANNs at CD dryer. According to the results, the coefficient of determination 0.998 and the RMSE value (0.0335) for the MR was obtained.

**Table 5:** The best values of evaluation criteria for FFBF and CFBF networks in different conditions of the number of layers and threshold functions for MR in CD dryer.

Network	Training algorithm	Threshold function	Number of layers and neurons	$MSE$	Train		Test	
					$R^{2}$	$MAE$	$R^{2}$	$MAE$
**FFBP**	**LM**	**TAN-TAN-PUR**	**2-10-10-1**	**0.00044**	**0.9993**	**0.0047**	**0.9994**	**0.0041**
FFBP	BR	TAN-LOG-PUR	2-15-10-1	0.00099	0.9988	0.0089	0.9990	0.0076
CFBP	LM	TAN-TAN-PUR	2-12-1	0.00084	0.9991	0.0064	0.9992	0.0057
CFBP	BR	TAN-TAN-TAN	2-8-8-1	0.00102	0.9988	0.0093	0.9989	0.0082

The best values of the evaluation criteria are shown in bold

#### Microwave dryer

Table 6 presents the best networks with the highest R^2^ values and the lowest MAE and MSE values for prediction MR of apple slices in MD dryer. The cascade forward back propagation (CFBP) structure, TAN-TAN-TAN threshold function, LM algorithm with 2–15-10–1 topology structure had the lowest $MSE$ (0.00059), $MAE$ (0.0045 for test and 0.0053 for train) values and the highest $R^{2}$ (0.9993 for test and 0.9991 train) values (Table 6).

**Table 6:** The best values of evaluation criteria for FFBF and CFBF networks in different conditions of the number of layers and threshold functions for MR in MD dryer.

Network	Training algorithm	Threshold function	Number of layers and neurons	$MSE$	Train		Test	
					$R^{2}$	$MAE$	$R^{2}$	$MAE$
FFBP	LM	TAN-PUR-LOG	2-5-5-1	0.00080	0.9988	0.0075	0.9989	0.0070
FFBP	BR	TAN-LOG-TAN	2-10-1	0.00064	0.9990	0.0059	0.9992	0.0051
**CFBP**	**LM**	**TAN-TAN-TAN**	**2-15-10-1**	**0.00059**	**0.9991**	**0.0053**	**0.9993**	**0.0045**
CFBP	BR	TAN-TAN-PUR	2-8-8-1	0.00095	0.9984	0.0094	0.9986	0.0088

The best values of the evaluation criteria are shown in bold

## Conclusion

The effects of CD and MD at different air temperatures (50, 60 and 70 °C), and microwave powers (90, 180, and 360 W) on the drying characteristics of apple slice were evaluated in this study. The drying time of apple slice was the lower in MD drying as compared to another one. Midilli et al. model was the most suitable model for prediction of apple MR. This model had the highest correlation coefficients ($R^{2}$) and lowest chi-square ($\chi^{2}$) and root mean square error (RMSE) values. So, it can be able to describe the thin layer drying characteristics of samples at two dryers. The maximum *D*_*eff*_ value of 8.21 × 10^−7^ m^2^/s was obtained under the MD with power of 360 W. The minimum SEC value (52.03 MJ/kg) was obtained from MD drying. The obtained R^2^ values using ANN for predication of MR at two different dryers (data test) were equal to 0.9993 and 0.9991 in CD and MD, respectively.

## List of symbols

$C_{pv}$: Vapor specific heat capacity (1004.16 J/(kg °C))
$C_{pa}$: Air specific heat capacity (1828.8 J/(kg °C))
$D_{eff}$: Effective moisture diffusivity (m^2^/s)
$$D_{0}$$: Constant number
$E_{a(c)}$: Energy of activation in CD method (kJ/mol)
$E_{a(m)}$: Energy of activation in MD method (W/g)
$h_{a}$: Absolute air humidity (kg_vapor_/kg_dry air_)
_*i*_: Positive integer
$L$: Half of the slab thickness (m)
M_e_: Equilibrium MC
M_0_: Initial MC (kg_water_/ kg_dry matter_)
$$MC$$: Moisture content (w.b)
$M$: Material MC (kg_water_/kg_dry matter_)
M_t_: MC at any time (kg_water_ /kg_dry matter_)
$$MR$$: Moisture ratio
$$MR_{\exp ,i}$$: Experimental value
$$MR_{pre,i}$$: Predicted value
$m_{w}$: Mass of evaporated water (kg)
$m_{v}$: Mass of removal water (kg)
$m$: Weight of raw sample (g)
*N*: Number of observations
*P*: Output power of microwave (W)
$P_{mic}$: Microwave output power (W)
$Q$: Inlet air to drying chamber (m^3^/min)
$R_{g}$: Universal gas constant (8.3143 kJ/mol)
$R^{2}$: Coefficient of determination
$RMSE$: Root mean square error
$SEC_{con}$: Specific energy consumption in CD method
$SEC_{mic}$: Specific energy consumption in MD method
$T_{in}$: Inlet air to drying chamber temperature (°C)
$T_{am}$: Ambient air temperature (°C)
$$t$$: Drying time (s)
$T_{a}$: Absolute air temperature (K)
$t_{2}$: Total drying time (min)
$$U_{n}$$: Normalized value
$$U_{R}$$: Actual value
$U_{\max }$: Maximum of the actual value
$U_{\min }$: Minimum of the actual value
$V_{h}$: Specific air volume (m^3^/kg)
$W$: Amount of evaporated moisture (g)
$W_{x}$: Initial weight of sample (g)
$W_{y}$: Dry matter content of sample (g)
*Z*: Constants
$\Delta E$: Total color differences
$$\Delta L*$$: Lightness difference
$\Delta a*$: Intensity of the color red
$\Delta b*$: Intensity of the color yellow
$$\chi^{2}$$: Chi square
